# Trends in Social Inequality in Overweight and Obesity Among Danish Infants, 2002-2022

**DOI:** 10.3389/ijph.2025.1608203

**Published:** 2025-08-18

**Authors:** Lis Marie Pommerencke, Sanne Ellegård Jørgensen, Sofie Weber Pant, Rikke Rothkegel Carlsson, Camilla Thørring Bonnesen, Lene Kierkegaard, Mette Rasmussen, Michael Davidsen, Trine Pagh Pedersen

**Affiliations:** National Institute of Public Health, Faculty of Health Sciences, University of Southern Denmark, Copenhagen, Denmark

**Keywords:** social inequality, infants, overweight and obesity, parental education, trend study

## Abstract

**Objectives:**

This study aimed to examine trends in relative and absolute social inequality in overweight/obesity among Danish infants born between 2002 and 2022.

**Methods:**

The study applied yearly cross-sectional data on infants’ weight and length at age 6–10 months, n = 63,100. Data was linked with parental education from population registers. Social inequality was measured by OR, relative index of inequality (RII), and slope index of inequality (SII). Trend tests measured changes over time.

**Results:**

No difference in odds for overweight/obesity by parental education was observed between 2002 and 2004, but from 2005 social inequality in overweight/obesity was revealed. The OR for overweight/obesity ranged from 1.20 (95% CI: 0.76–1.89) to 2.31 (95% CI: 1.64–3.25) for infants of parents with lowest educational attainment. RII ranged from 0.78 to 0.41 (test for trend, p = 0.179) and SII ranged from −0.92 to −4.54 (test for trend, p = 0.026) indicating a persistent relative social inequality and an increase in absolute social inequality in overweight/obesity from 2002 to 2022, respectively.

**Conclusion:**

The study revealed persistent relative social inequality and increased absolute social inequality in overweight/obesity among Danish infants from 2002 to 2022.

## Introduction

Childhood overweight and obesity are a global health challenge [1, 2]. There have been indications of stagnating prevalence levels in the US, Australia, and Europe [3–5], including Denmark [6]. However, the prevalence levels are still high [1]. It is widely recognized that increased body mass index (BMI) in infancy is a predictor of overweight and obesity though childhood, adolescence, and into adulthood [7–9]. This tracking pattern has also been observed in Danish cohorts [10, 11].

Childhood overweight and obesity are unevenly distributed across parental socioeconomic position (SEP) with a significantly higher proportion of children being overweight or obese among parents with lower SEP [12, 13]. Social inequality in child health emerges through a range of complex and interrelated pathways, which are largely driven by the economic, material, and psychosocial conditions in which children grow up [14]. Social inequality is present already in the first year of life, as nutritional factors such as duration of breastfeeding and dietary intake, as well as maternal BMI, which are known to be associated with risk of childhood overweight and obesity, are unevenly distributed across SEP groups [9, 15–17]. Few studies have found similar social patterns in height, weight, and BMI in the first year of life [18, 19]. A Danish study including data from 1997 to 2003 found that infants of parents with low educational level have higher risk of low BMI at birth until the age of 5 months than infants of parents with high educational level. Hereafter, the social gradient levels out until the age of 12 months followed by an emerging social gradient characterized by an increased risk of overweight and obesity among children of parents with low educational level compared to children of parents with high educational level [20]. Trends in social inequality in childhood overweight and obesity have been described for different age groups, and across studies no consistent conclusions on trends in social inequality patterns can be drawn [12, 21, 22]. A review of 30 studies (children aged 2–18) found based on absolute measures of inequality that half of studies indicated an increasing gap in overweight and obesity between SEP groups measured by different indicators e.g., parental education, parental income, neighborhood deprivation and school SEP [12]. Post year 2000 stagnation or improvements in overweight and obesity prevalence were less apparent among low SEP children compared to high SEP children. Additionally, 40% of these studies reported a widening of SEP inequalities in overweight and obesity [12]. To our knowledge, no trend studies on infant overweight and obesity across SEP have been published.

From a public health perspective, it is important continuously to monitor developments in prevalence levels over time to evaluate whether health promotion initiatives have reached the intended effect. Additionally, overweight and obesity are unevenly distributed within populations, and trends at the general population level may not fully reflect patterns in different subgroups. Therefore, it is important that trends in overweight and obesity are illustrated for groups of varying risk. Inequality in health is often described using absolute or relative measures. Absolute inequality is the absolute difference in a health indicator, e.g., overweight prevalence, between subgroups, calculated by subtracting one group’s mean from the other’s, while relative inequality is the ratio between subgroups, calculated by dividing one group’s mean by the other’s [23]. Thus, absolute inequality reflects the magnitude of the difference, whereas relative inequality reflects the proportional difference [23]. When studying trends in inequalities among subgroups, conclusions may vary depending on whether absolute or relative measures of inequality are applied [24]. For example, if prevalence increases in all subgroups, the absolute difference may increase, whereas the relative difference decreases because the increase in the absolute difference is not large enough to also increase the relative difference [24]. Furthermore, the importance of using both measures is especially relevant within a public health setting, where estimation of the absolute social inequality might be more relevant as it indicates the magnitude of the public health challenge by reflecting the absolute number of children from lower socioeconomic strata who have overweight or obesity. A high relative social inequality in overweight and obesity may matter less in a public health context if the overall prevalence of overweight and obesity is low [21].

The aim of this study is to examine trends in social inequality in overweight and obesity among Danish infants born between 2002 and 2022 using both absolute and relative measures of inequality.

## Methods

### Setting

This study was conducted in Denmark with data collected yearly in the period 2002–2022. In Denmark, every family with a newborn child is offered a series of free home visits from a community health nurse. A community health nurse is a nurse with a 1.5-year further education in child health and development. The community health nurses visit approximately 95% of all families with a newborn in Denmark [25]. The Danish Health Authority recommends five home visits during the first year. At these visits, the community health nurses systematically register data on child health and development including weight and length.

This study is based on data from the Child Health Database, which is a collaboration between community health nurses from municipalities in Denmark. The collaboration was initiated by community health nurses in 2002 where 16 municipalities participated and since 2015 33 of the 98 municipalities in Denmark have been a part of the database. Eight municipalities have participated with data on length and weight throughout the entire study period (2002–2022). These eight municipalities are mainly located in suburban Copenhagen and comprise citizens from diverse socioeconomic backgrounds. A Danish report from 2020 shows that the prevalence of infant overweight and obesity in these eight municipalities is similar to the national average [26]. In the Child Health Database collaboration, data collected by community health nurses were registered based on the application of a common manual, securing comparability, and increasing validity of the records. Two record systems, Solteq Sund and Novax, were used to collect the data. All Danish citizens receive a unique personal identification number at birth, enabling exact individual-level linkage between national registers in Denmark as well as linking the infant’s identification number with its parents’ identification numbers. The two record systems, Solteq Sund and Novax, included the infant’s unique personal identification number, by which it was possible to link the data to Danish registers. Data was linked with national health and sociodemographic registers with complete data from the entire population.

### Study Design and Study Population

The study design involved continuous and yearly cross-sectional data collections among infants from birth to 10 months. Infants enrolled in the study were born between 1st January 2002 and 31st December 2022 and included all infants from the eight municipalities with data on weight and length in the Child Health Database collaboration. The specific study population for this study was infants aged 6–10 months. The age range of 6–10 months was chosen because, at this stage infants in Denmark are no longer fully breast-fed or fully bottle-fed and begin to become more active. A total of 77,245 infants from the eight municipalities were registered, of which 63,100 (81.7%) had complete data on weight and length. Missing data analyses showed that infants with missing data on weight and length were more likely to not live with both parents, have mothers under the age of 30, have unemployed parents, and have parents with non-Danish origin (data not shown). Despite being statistically significant, the differences were relatively minor and are therefore not expected to substantially impact the main findings.

### Measure of Exposure

Different indicators of SEP, such as neighborhood deprivation, parental income, educational attainment of the parents, and parental employment, have been used for studying social inequalities in childhood overweight and obesity. Parental income and/or education are recommended [27], and in Denmark, these factors are closely related. In this study, we applied parental education because we study trends over time and education tends to change more slowly compared to income, which can fluctuate more in a short period. Furthermore, education may influence cognitive functioning, making a person more receptive to health education messages, and better equipped to access and communicate with health services [28]. Parental education was measured by parents’ highest ongoing or completed educational attainment at the infant’s birth, and this information was retrieved from the Population’s Educational Register from Statistics Denmark [29]. The highest educational attainment of the parents was categorized into five hierarchical levels from the highest level of education (5 or more completed years at university) to the lowest level (primary school, 9 years at school). Infants were categorized according to the highest educational level of their parents. If the education level of only one parent was available, it was used to categorize the infant.

### Measure of Outcome

Measurements of weight and recumbent length between 6–10 months were assessed by the community health nurse. The infant was weighed without diaper and only light clothes using a handheld hanging scale. Recumbent length was measured on either a measuring board or with measuring tape. The Danish Health Authority recommends the weight to be rounded to one decimal place and the recumbent length to be measured to nearest half centimeter [30]. The outcome estimate of BMI status was based on weight and length measured at age 6–10 months combined with information on infant sex and age. A recent article recommends using BMI z-scores from birth and forward [31]. BMI z-scores were calculated using sex- and age-specific standard values from the WHO [32]. Infants at age 6–10 months were classified as overweight, when BMI z-score was between +2 SD and +3 SD and obese, when BMI z-score was greater than +3 SD.

### Co-Variates

Demographic information on infant sex, maternal and paternal age at the time of childbirth (<20, 20–29, 30–39, and ≥40), parental origin (two, one, or no parents of Danish origin), parental occupational attachment (two, one, or no parents in work or education), as well as family type (infant lives with both parents, yes vs. no) were included to describe the characteristics of the study population. Information on these variables were sourced from national population registers: the Danish Civil Registration System [33] and the Employment Classification Module [34].

### Statistical Analyses

The statistical analyses were conducted in SAS version 9.4. In all analyses, level of significance was set to p < 0.05. The study period 2002–2022 was divided into seven time periods, each consisting of 3 years. Cochran-Armitage test for trend was used to assess trends in prevalence of overweight and obesity from 2002 to 2022. Prevalence differences within each of the seven time periods were tested with chi-square test. Both relative and absolute social inequality are reported [24]. To estimate the relative social inequality for each educational level within the seven time periods, we calculated odds ratio (OR) for overweight and obesity with simple logistic regression analysis with highest level of education as reference group. To account for different distribution of parental educational attainment within and across the time periods, both Slope Index of Inequality (SII) and Relative Index of Inequality (RII) were calculated. SII predicts the difference between the top and bottom of the social hierarchy, (e.g., those with the highest and lowest level of education), of a health indicator, whereas RII predicts the ratio [23, 35]. Both measures are obtained by regression-based weighted analysis. For SII, the dependent variable (the outcome variable: “overweight/obesity at 6–10 months” weighted by the population in each group) was regressed against the midpoint scores of the five educational levels within each of the seven time periods. The RII was estimated similarly, with the exception that the dependent variable in the regression model was logit-transformed, followed by taking the exponential function of the parameter estimate [23, 36]. SII positive values indicate that infant overweight/obesity is more prevalent among the more educated groups, negative values indicate that infant overweight/obesity is more prevalent among the less educated groups, and zero indicates no inequality [23, 36]. Values of the RII greater than one indicate a higher prevalence in the most educated groups and values less than one illustrate a higher prevalence in the less educated groups [23]. Lastly, we conducted trend analyses to test for change in social inequality over time. Trend test reports whether the changes in SII and RII over time are stable or there has been a significant change. Trend test was performed by a simple linear regression of SII and RII.

### Data Protection and Ethical Considerations

The data collection was approved by the Research and Innovation Organization at the University of Southern Denmark (registration number 11,667) and the Danish Regional Council (registration number R-22030405) and complies with national regulations of data protection and consent. Data was passed on from the municipalities to the National Institute of Public Health as described in the Data Protection Legislation [37] and comply with the Danish codex for integrity in research [38].

## Results

### Sociodemographic Characteristics

Table 1 provides an overview of the distribution of sociodemographic characteristics of the study population. A total of 63,100 infants born between 2002 and 2022 were enrolled in the study. The total study population was characterized by 62% of the infants’ parents having a higher education or ≥5 years of university education, 82% of the parents were in job or pursuing education, and 64% of the infants had two parents of Danish origin. About 55% of the infants were born by parents being 30–39 years old at the time of childbirth and 90% lived with both parents. During the study period (2002–2022), a clear shift in the distribution of parental educational attainment occurred with an increasing proportion of parents having a ≥5 years university education. Minor variations in the distribution of the other sociodemographic variables were observed across the study period.

**TABLE 1:** Description of study population (Denmark, 2002–2022).

	Time periods							
	2002–2004 N = 7,637	2005–2007 N = 8,651	2008–2010 N = 9,357	2011–2013 N = 8,909	2014–2016 N = 9,679	2017–2019 N = 9,535	2020–2022 N = 9,332	Total N = 63,100
	n (%)	n (%)	n (%)	n (%)	n (%)	n (%)	n (%)	n (%)
**Infant sex**								
Girl	3,761 (49.3)	4,255 (49.2)	4,504 (48.1)	4,393 (49.3)	4,687 (48.4)	4,678 (49.1)	4,514 (48.4)	30,792 (48.8)
Boy	3,876 (50.8)	4,396 (50.8)	4,853 (51.9)	4,516 (50.7)	4,992 (51.6)	4,857 (50.9)	4,818 (51.6)	32,308 (51.2)
**Maternal age**								
<20 years	102 (1.3)	123 (1.4)	121 (1.3)	90 (1.0)	57 (0.6)	45 (0.5)	31 (0.3)	569 (0.9)
20–29 years	3,349 (44.0)	3,480 (40.4)	3,607 (38.7)	3,408 (38.5)	3,828 (39.8)	3,744 (39.5)	3,482 (37.5)	24,898 (39.7)
30–39 years	3,944 (51.8)	4,676 (54.3)	5,169 (55.5)	4,937 (55.8)	5,238 (54.5)	5,198 (54.9)	5,311 (57.2)	34,473 (54.9)
40 years or older	223 (2.9)	334 (3.9)	420 (4.5)	419 (4.7)	494 (5.1)	489 (5.2)	468 (5.0)	2,847 (4.5)
**Paternal age**								
<20 years	19 (0.3)	40 (0.5)	41 (0.5)	24 (0.3)	19 (0.2)	6 (0.1)	9 (0.1)	158 (0.3)
20–29 years	2,191 (29.1)	2,238 (26.3)	2,371 (26.0)	2,254 (26.0)	2,450 (26.1)	2,482 (26.8)	2,175 (24.1)	16,161 (26.3)
30–39 years	4,429 (58.9)	5,042 (59.3)	5,363 (58.7)	5,025 (58.1)	5,337 (56.9)	5,345 (57.6)	5,393 (59.6)	35,934 (58.4)
40 years or older	886 (11.8)	1,180 (13.9)	1,361 (14.9)	1,352 (15.6)	1,580 (16.8)	1,441 (15.5)	1,468 (16.2)	9,268 (15.1)
**Parental origin**								
Two parents of Danish origin	5,531 (72.4)	6,311 (73.0)	6,432 (68.7)	5,753 (64.6)	5,754 (59.5)	5,385 (56.5)	5,146 (55.1)	40,312 (63.9)
One parent of Danish origin	655 (8.6)	813 (9.4)	1,012 (10.8)	1,045 (11.7)	1,232 (12.7)	1,257 (13.2)	1,331 (14.3)	7,345 (11.6)
No parents of Danish origin	1,451 (19.0)	1,527 (17.7)	1,913 (20.4)	2,110 (23.7)	2,693 (27.8)	2,893 (30.3)	2,855 (30.6)	15,442 (24.5)
**Parental highest education**								
Primary school	709 (9.3)	776 (9.0)	986 (10.5)	812 (9.1)	787 (8.1)	596 (6.3)	428 (4.6)	5,094 (8.1)
Vocational education	2,305 (30.2)	2,230 (25.8)	2,247 (24.0)	2,130 (23.9)	2,054 (21.2)	1,818 (19.1)	1,434 (15.4)	14,218 (22.5)
High school education	712 (9.3)	759 (8.8)	737 (7.9)	670 (7.5)	702 (7.3)	650 (6.8)	533 (5.7)	4,763 (7.6)
Higher education	2,041 (26.7)	2,526 (29.2)	2,513 (26.9)	2,517 (28.3)	2,737 (28.3)	2,974 (31.2)	2,969 (31.8)	18,277 (29.0)
≥5 years of university education	1,870 (24.5)	2,360 (27.3)	2,874 (30.7)	2,780 (31.2)	3,399 (35.1)	3,497 (36.7)	3,968 (42.5)	20,748 (32.9)
**Parental occupational attachment**								
Two parents in work or education	6,047 (79.8)	6,991 (81.5)	7,887 (85.1)	7,145 (81.3)	7,566 (79.3)	7,619 (81.0)	7,756 (84.0)	51,011 (81.8)
One parent in work or education	1,185 (15.6)	1,295 (15.1)	1,150 (12.4)	1,379 (15.7)	1,604 (16.8)	1,541 (16.4)	1,326 (14.4)	9,480 (15.2)
No parents in work or education	350 (4.6)	288 (3.4)	229 (2.5)	265 (3.0)	369 (3.9)	249 (2.7)	155 (1.7)	1,905 (3.1)
**Family type**								
Lives with both parents	7,006 (91.9)	7,869 (91.2)	8,394 (89.9)	7,992 (90.0)	8,646 (89.6)	8,578 (90.2)	8,430 (90.5)	56,915 (90.4)
Does not live with both parents	622 (8.1)	761 (8.8)	941 (10.1)	886 (10.0)	999 (10.4)	930 (9.8)	881 (9.5)	6,020 (9.6)

### Infants With Overweight or Obesity at 6–10 Months of Age

Table 2 shows the prevalence of infants with overweight or obesity at age 6–10 months across levels of parental educational attainment by time period, as well as OR (95% CI) for overweight and obesity across levels of parental educational attainment. Table 3 shows RII and SII for overweight and obesity by time period and trend test of RII and SII.

**TABLE 2:** Proportion and odds ratio (OR) (95% CI) of infants with overweight/obesity at 6–10 months of age by highest educational attainment of the parents in 2002–2022 (Denmark, 2002–2022).

	Time periods													
	2002–2004 N = 7,637		2005–2007 N = 8,651		2008–2010 N = 9,357		2011–2013 N = 8,909		2014–2016 N = 9,679		2017–2019 N = 9,535		2020–2022 N = 9,332	
	n (%)	OR^a^ (95% CI)	n (%)	OR^a^ (95% CI)	n (%)	OR^a^ (95% CI)	n (%)	OR^a^ (95% CI)	n (%)	OR^a^ (95% CI)	n (%)	OR^a^ (95% CI)	n (%)	OR^a^ (95% CI)
Primary school	28 (4.0)	1.20 (0.76; 1.89)	41 (5.3)	**1.61 (1.10; 2.37)**	62 (6.3)	**1.92 (1.39; 2.67)**	47 (5.8)	**1.95 (1.35; 2.81)**	59 (7.5)	**1.79 (1.31; 2.45)**	48 (8.1)	**2.31 (1.64;** **3.25)**	27 (6.3)	1.26 (0.83; 1.90)
Vocational education	91 (4.0)	1.20 (0.86; 1.67)	94 (4.2)	1.27 (0.94; 1.72)	101 (4.5)	**1.35 (1.01; 1.79)**	108 (5.1)	**1.69 (1.27; 2.26)**	129 (6.3)	**1.48 (1.16; 1.89)**	111 (6.1)	**1.71 (1.32; 2.22)**	113 (7.9)	**1.60 (1.26; 2.03)**
High school education	21 (3.0)	0.89 (0.54; 1.47)	26 (3.4)	1.02 (0.65; 1.61)	24 (3.3)	0.96 (0.61; 1.52)	27 (4.0)	1.33 (0.86; 2.07)	43 (6.1)	**1.44 (1.02; 2.05)**	44 (6.8)	**1.91 (1.34; 2.72)**	22 (4.1)	0.80 (0.51; 1.26)
Higher education	68 (3.3)	1.01 (0.71; 1.43)	89 (3.5)	1.05 (0.78; 1.44)	92 (3.7)	1.09 (0.81; 1.45)	114 (4.5)	**1.50 (1.13; 2.00)**	127 (4.6)	1.08 (0.84; 1.37)	156 (5.3)	**1.46 (1.15; 1.85)**	192 (6.5)	**1.29 (1.05; 1.58)**
≥5 years of university education	62 (3.3)	1	79 (3.4)	1	97 (3.4)	1	85 (3.1)	1	147 (4.3)	1	128 (3.7)	1	202 (5.1)	1
All^b^	270 (3.5)		329 (3.8)		376 (4.0)^*^		381 (4.3)^*^		505 (5.2)^*^		487 (5.1)^*^		556 (6.0)^*^	

n = Number of children with overweight/obesity at 6–10 months.

^*^ Indicates statistically significant difference in prevalence of overweight/obesity (p < 0.05) between parental educational levels within the seven time periods tested using chi-square test.

^a^ Unadjusted OR.

^b^ Cochran-Armitage test for trend was used to assess trends in the overall prevalence of overweight and obesity from 2002 to 2022 (p < 0.001).

Bold values indicate statistical significance at p < 0.05.

**TABLE 3:** Relative index of inequality (RII) and slope index of inequality (SII) of infants with overweight/obesity at 6–10 months of age by highest educational attainment of the parents in 2002–2022 and trend test of RII and SII (Denmark, 2002–2022).

	Time periods							
	2002–2004	2005–2007	2008–2010	2011–2013	2014–2016	2017–2019	2020–2022	Trend test^a^
RII^b^ (95% CI)	0.78 (0.51; 1.19)	**0.64 (0.43; 0.96)**	0.55 (0.27; 1.11)	**0.45 (0.27; 0.77)**	**0.53 (0.35; 0.78)**	**0.41 (0.28; 0.59)**	0.63 (0.26; 1.52)	Est. = −0.03 (−0.09; 0.02) p = 0.179
SII^c^ (95% CI)	−0.92 (−2.37; 0.53)	−1.79 (−3.63; 0.06)	−2.70 (−6.22; 0.82)	**−3.26 (−5.26; −1.27)**	**−3.51 (−5.95; −1.07)**	**−4.54 (−6.80; −2.27)**	−2.95 (−7.98; 2.07)	**Est. = −0.44 (−0.81; −0.08) p = 0.026**

^a^ Trend test by a simple linear regression of SII and RII.

^b^ RII measures the relative difference in the proportion of infants with overweight/obesity at 6–10 months between lowest and highest parental education level. Values of the RII greater than one indicate a higher prevalence in the most educated groups and values less than one illustrate a higher prevalence in the less educated groups.

^c^ SII measures the absolute difference in the proportion of infants with overweight/obesity at 6–10 months between lowest and highest parental education level. Positive values of SII indicate that infant overweight/obesity is more prevalent among the more educated groups, while negative values indicate that infant overweight/obesity is more prevalent among the less educated groups, and zero indicates no inequality.

Bold values indicate statistical significance at p < 0.05.

#### Prevalence of Overweight and Obesity

**FIGURE 1:**
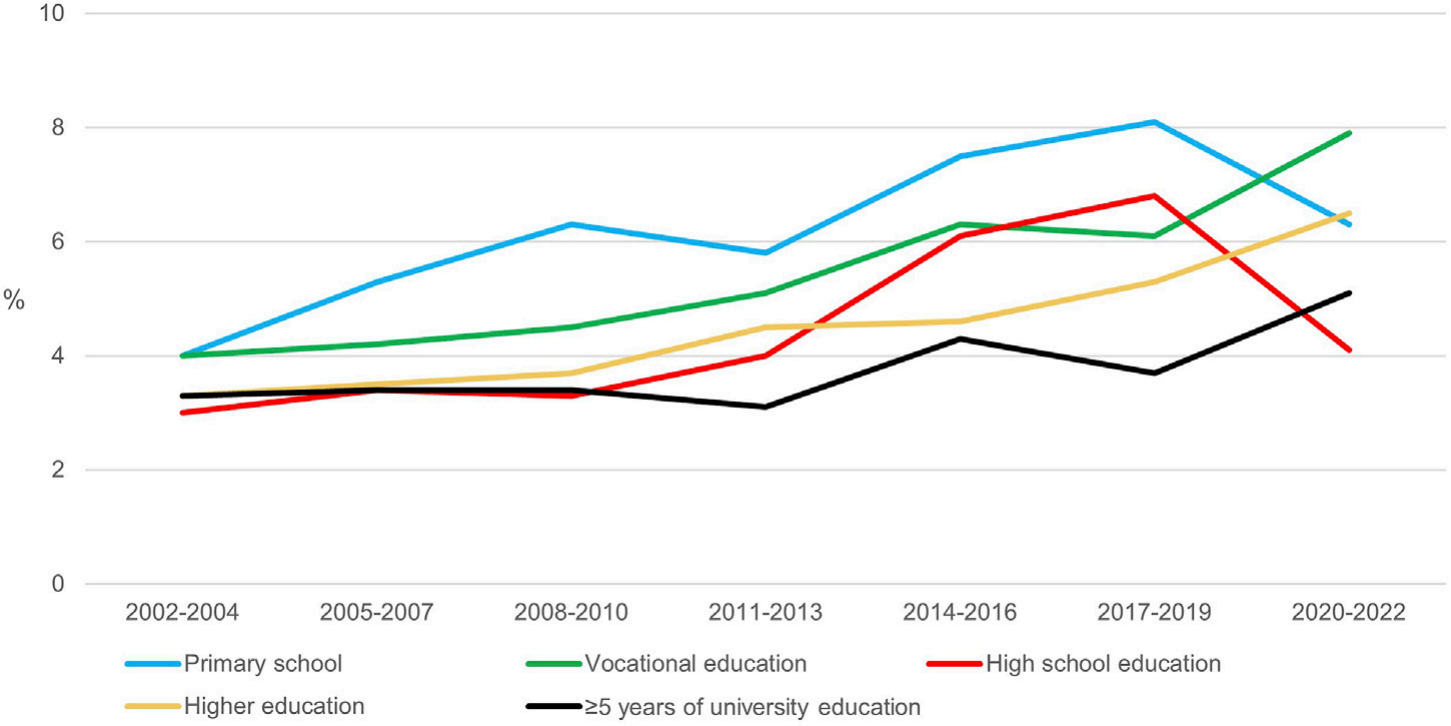
Proportion of infants with overweight/obesity at 6–10 months of age by highest educational attainment of the parents in 2002–2022 (Denmark, 2002–2022).

From 2002 to 2022, the overall prevalence of infants with overweight or obesity at age 6–10 months increased from 3.5% to 6.0% (test for trend, p < 0.001). During the time period from 2002 to 2007, no significant differences were observed in the prevalence of overweight and obesity across levels of parental educational attainment. This contrasts with the period from 2008 to 2022 where significant differences in the prevalence of overweight and obesity across levels of parental educational attainment were observed. From 2008 to 2018, infants of parents with primary school had the highest prevalence of overweight and obesity, while those with parents with ≥5 years of university education had the lowest. In the most recent time period (2020–2022), the prevalence of overweight and obesity across levels of parental educational attainment slightly shifted. Infants of parents with vocational education showed the highest prevalence (7.9%), whereas infants of parents with high school had the lowest prevalence (4.1%). These trends are illustrated in Figure 1, highlighting a notable increase in overweight and obesity among “primary education” from 2005, whereas “vocational education,” “high school education” and “higher education” showed less increases in overweight and obesity from 2008.

#### Relative Social Inequality in Overweight and Obesity

The differences in prevalence of overweight and obesity across educational levels were supported by the OR measuring relative social inequality. No statistically significant difference in odds for overweight and obesity across levels of parental educational attainment were observed in 2002–2004. However, from 2005 to 2022 social inequality in overweight and obesity at age 6–10 months was revealed. For example, from 2005 to 2019 the OR for overweight and obesity ranged from 1.61 (95% CI: 1.10–2.37) to 2.31 (95% CI: 1.64–3.25) for infants of parents with lowest level of educational attainment compared to infants of parents with highest educational attainment.

The RII for overweight and obesity at age 6–10 months across the seven time periods are presented in Table 3 and ranged from 0.78 to 0.41 during the study period being 0.78 in 2002–2004 and 0.63 in 2020–2022. The RII was smaller than 1 in all time periods and statistically significant in 2005–2007, 2011–2013, 2014–2016 and 2017–2019. A test for trend of the RII (p = 0.179) revealed no significant change in relative social inequality in overweight and obesity from 2002 to 2022.

#### Absolute Social Inequality in Overweight and Obesity

The SII for overweight and obesity at age 6–10 months in each time period presented in Table 3 were less than zero in all periods and statistically significant in 2011–2013, 2014–2016 and 2017–2019. SII ranged from −0.92 to −4.54 during the entire study period with a value of −0.92 in 2002–2004 and −2.95 in 2020–2022. Test for trend showed a significant (p = 0.026) decrease in SII from 2002 to 2022 suggesting an overall increase in absolute social inequality in overweight and obesity from 2002 to 2022.

## Discussion

### Main Findings

The present study aimed to investigate trends in social inequality in infant overweight and obesity between 2002 and 2022. The study revealed no absolute or relative social inequality between 2002 and 2004. From 2005 to 2019, we found social inequality in overweight and obesity with increasing odds for overweight and obesity among infants of parents with lower educational attainment. However, changes occurred in 2020–2022 where increased odds for overweight and obesity were only observed for infants of parents with vocational education and higher education. Across the entire study period (2002–2022), we found an increased absolute social inequality (measured by SII) in overweight and obesity among infants aged 6–10 months, whereas no upward or downward trend was observed for the relative social inequality (measured by RII) in overweight and obesity. To our knowledge, no previous studies have investigated trends in social inequality in infant overweight and obesity. Previous studies have investigated trends in social inequality in childhood overweight and obesity (aged >2 years) showing a persistent social inequality in overweight and obesity over time [21, 22]. Further, some studies have found an increasing prevalence or less improvements in prevalence of overweight and obesity among children with low SEP, whereas levelling off or improved overweight and obesity prevalence are found in children with high SEP [12, 39].

The present study found an overall increasing prevalence of infants with overweight and obesity from 2002 to 2022. These findings are not in line with existing literature showing a stagnating prevalence in overweight and obesity among infants in the US and Denmark [5, 6]. This discrepancy might be caused by different time periods studied. The present study covered the period from 2002 to 2022, whereas the two other studies followed infants until 2011 and 2012, respectively [5, 6].

It is important to highlight that the most recent time period (2020–2022) in this study was influenced by the COVID-19 lockdowns. During this period, the prevalence of overweight and obesity decreased for some educational levels, while it increased for others. The reasons for these changes are difficult to interpret. Visits from community health nurses may have been canceled, which potentially have affected the observed changes in prevalence of overweight and obesity. However, other underlying factors may also have an impact as fluctuating prevalence of overweight and obesity within each educational level was observed in other time periods, such as among infants of parents with primary school between time period 2008–2010 and 2011–2013. However, it is relevant to closely examine how the COVID-19 lockdowns may have affected the prevalence of overweight and obesity, and to identify the factors contributing to variations in prevalence across different levels of educational attainment. Supplementary analyses excluding the most recent time period including the COVID-19 lockdowns (2020–2022) were performed. These analyses showed a significant decrease in both RII and SII from 2002 to 2019, suggesting an increase in relative and absolute social inequality in overweight and obesity among infants from 2002 to 2019. These findings emphasize the need for further research on how the social inequality in overweight and obesity among infants will develop in the coming years. Furthermore, given that social inequality in child health emerges through a wide range of complex pathways [14], it would be interesting to explore how other socioeconomic factors, such as parental origin or parental occupational status, influence the development of overweight and obesity.

The RII and SII led to different conclusions which may be explained by the sensitivities of the two measures. During the study period, the overall prevalence of overweight and obesity increased and there was a shift in the distribution of parental educational attainment, with an increasing proportion of parents having a ≥5 years university education. Also, minor variations occurred in the distribution of the other sociodemographic variables. For example, a small increase in infants with no parents of Danish origin was observed. The SII is sensitive to changes in population distribution among different socioeconomic categories as well as to changes in the frequency of the health problem being studied [36]. For example, if the overall health level improves proportionately in all the socioeconomic categories, the SII will increase, while relative differences will remain constant [36]. The RII is sensitive to changes in educational attainment levels. It may show larger value in one population compared to another (e.g., across different time periods) due to a larger inequality in the distribution of individuals across the socioeconomic categories [36]. Also, the RII may decrease when the health problem being studied improves due to an upward shift in the educational levels [40]. Renard et al. [40] cautions against using RII and SII for assessing inequality when socioeconomic structures change suggesting instead showing the absolute changes within the socioeconomic categories. We therefore chose to present both the absolute prevalence of overweight and obesity for each educational attainment level as well as OR for each educational level along with RII and SII.

A large number of interventions aiming to prevent overweight and obesity in children have been developed. However, only a small number of studies have focused on weight development in infancy [41], and few have shown to be efficient in reducing social inequalities in early childhood overweight/obesity [42]. The lack of effective interventions may be caused by a limited understanding of the complexity of factors contributing to social inequality in overweight and obesity. It is proposed that children and adolescents develop obesity through interactions of genetic, behavioural, psychosocial, and environmental factors, interactions which are all influenced by SEP [43, 44]. In a new model for obesity development, Hemmingsson and colleagues (2023) explores the complex interplay of various factors contributing to obesity. For example, the model emphasizes that prolonged social adversity within the family (e.g., parents having a low education, low social position, poverty or uncertain accommodation) increases the risk of parental chronic stress and psychological strain which in turn contribute to an insecure and disharmonious family environment. According to the model, failures to meet the child’s needs may lead to disrupted energy balance homeostasis, resulting in weight gain and obesity [43], a situation that might be more prevalent in low SEP families. The higher absolute number of infants with overweight or obesity among parents with high levels of educational attainment compared to infants of parents with lower educational attainment underline the need for universal prevention rather than solely relying on interventions targeted at high-risk groups. A recent Danish review of the international literature concludes that more comprehensive and multicomponent interventions are needed to address the challenge of childhood overweight and obesity [41, 45]. Many of the previous interventions have followed a one-size-fits all approach, but it is likely that different target groups (e.g., fathers/partners, families of low SEP, and ethnic minorities) need different modes of delivery [46].

### Strengths and Limitations

The present study population included almost complete populations of infants in eight Danish municipalities affiliated to the Child Health Database collaboration with data on weight and length during the entire study period (2002–2022). The exposure variable combined the level of education of both parents, categorizing the infant by the parent with the highest educational level. We chose this approach because, in most families in Denmark, both parents are highly involved in raising children, and the duration of paternity leave has increased in recent years. While women tend to have slightly higher levels of education [47], men still earn more than women in comparable similar job positions [48]. By linking community health nurses’ records with national population registers this study had high level of data coverage and is thus less likely subject to selection bias. Given the extensive experience and training of the community health nurses, we expect their measurements of weight and recumbent length to be less prone to measurements errors, compared to those reported by parents. We chose to use BMI z-scores instead of weight for length z-scores. In a recent publication, Roberge and colleagues found no substantial differences between weight for length z-scores and BMI z-scores in predictive efficacy of adiposity and cardiometabolic measures [31]. Thus, they advocate for the convenience of using the BMI z-scores from birth eliminating the need to switch between methods when reaching the age of 2 years where BMI z-scores are the recommended standard [31]. Further, in contrast to weight for length, BMI reflects age-dependent variations in weight and length during infancy and can be adjusted for gestational age [31].

The missing analyses suggest that the analyses might have underestimated the prevalence of overweight and obesity and have weakened the correlation between exposure and outcome. This inference arises from the observation that the infants with incomplete data were characterized by for example, being more likely to not live with both parents and having unemployed parents and these factors might serve as risk factors for childhood overweight [49]. The study population covers only eight out of the 98 municipalities in Denmark, with most of these eight municipalities located in suburban Copenhagen and the generalizability of the study may therefore be limited. However, a Danish report from 2020 observed that the prevalence of infant overweight and obesity in the eight municipalities, which were included in the present study, was similar with the national prevalence [26].

### Implications

Within current efforts towards reducing the risk of developing childhood overweight or obesity, there is generally a concurrent and strong focus on reducing social inequalities [41]. Continued monitoring of changes in both prevalence levels and social inequalities in infant and child overweight and obesity are therefore essential. From these indications of the efficiency of especially population level actions and changes, initiatives and interventions can be drawn. It is therefore essential that future overweight and obesity prevention is accompanied with prioritized monitoring initiatives.

### Conclusion

From 2002 to 2022, the prevalence of infants with overweight or obesity at age 6–10 months increased. No relative social inequality was observed in 2002–2004, but from 2005 to 2019 relative social inequality in overweight and obesity with increasing odds for overweight and obesity among infants of parents with lower educational attainment were found. The relative index of inequality (RII) suggested a persistent relative social inequality in overweight and obesity among infants aged 6–10 months from 2002 to 2022, however, the slope index of inequality (SII) indicated an increase in absolute social inequality in overweight and obesity from 2002 to 2022.
